# Infarctions in the vascular territory of the posterior cerebral artery: clinical features in 232 patients

**DOI:** 10.1186/1756-0500-4-329

**Published:** 2011-09-07

**Authors:** Adrià Arboix, Guillermo Arbe, Luis García-Eroles, Montserrat Oliveres, Olga Parra, Joan Massons

**Affiliations:** 1 Unit of Cerebrovascular Diseases, Service of Neurology, Hospital Universitari del Sagrat Cor, Universitat de Barcelona, Barcelona, Spain; 2 CIBER de Enfermedades Respiratorias (CB06/06), Instituto Carlos III, Madrid, Spain; 3 Unit of Organization, Planning and Information Systems, Consorci Sanitari del Maresme, Barcelona, Spain; 4 Service of Pneumology, Hospital Universitari del Sagrat Cor, Universitat de Barcelona, Barcelona, Spain

## Abstract

**Background:**

Ischemic stroke caused by infarction in the territory of the posterior cerebral artery (PCA) has not been studied as extensively as infarctions in other vascular territories. This single centre, retrospective clinical study was conducted a) to describe salient characteristics of stroke patients with PCA infarction, b) to compare data of these patients with those with ischaemic stroke due to middle cerebral artery (MCA) and anterior cerebral artery (ACA) infarctions, and c) to identify predictors of PCA stroke.

**Findings:**

A total of 232 patients with PCA stroke were included in the "Sagrat Cor Hospital of Barcelona Stroke Registry" during a period of 19 years (1986-2004). Data from stroke patients are entered in the stroke registry following a standardized protocol with 161 items regarding demographics, risk factors, clinical features, laboratory and neuroimaging data, complications and outcome. The characteristics of these 232 patients with PCA stroke were compared with those of the 1355 patients with MCA infarctions and 51 patients with ACA infarctions included in the registry.

Infarctions of the PCA accounted for 6.8% of all cases of stroke (*n *= 3808) and 9.6% of cerebral infarctions (*n *= 2704). Lacunar infarction was the most frequent stroke subtype (34.5%) followed by atherothrombotic infarction (29.3%) and cardioembolic infarction (21.6%). In-hospital mortality was 3.9% (*n *= 9). Forty-five patients (19.4%) were symptom-free at hospital discharge. Hemianopia (odds ratio [OR] = 6.43), lacunar stroke subtype (OR = 2.18), symptom-free at discharge (OR = 1.92), limb weakness (OR = 0.10), speech disorders (OR = 0.33) and cardioembolism (OR = 0.65) were independent variables of PCA stroke in comparison with MCA infarction, whereas sensory deficit (OR = 2.36), limb weakness (OR = 0.11) and cardioembolism as stroke mechanism (OR = 0.43) were independent variables associated with PCA stroke in comparison with ACA infarction.

**Conclusions:**

Lacunar stroke is the main subtype of infarction occurring in the PCA territory. Several clinical features are more frequent in stroke patients with PCA infarction than in patients with ischaemic stroke due to infarction in the MCA and ACA territories. In-hospital mortality in patients with PCA territory is low.

## Background

Knowledge of the clinical features of cerebral infarctions according to the affected vascular territory is important to characterize the diverse spectrum of symptoms associated with the vascular topography of lesions. Infarctions in the territory of the posterior cerebral artery (PCA) are not uncommon [1] but some aspects of the natural history of PCA infarction, such as aetiology, clinical features and outcome have not been sufficiently documented. In many studies, patients with PCA infarction are included in group of hemispheric cerebral infarction as a whole, independently of the different vascular topography of lesions [1-3]. Moreover, the differential clinical profile between ischaemic stroke caused by PCA infarctions, middle cerebral artery (MCA) infarctions and anterior cerebral artery (ACA) infarctions is poorly defined, probably because separate analysis of PCA stroke as an individual clinical entity is rarely performed.

This single centre, retrospective clinical study was conducted with the following aims: a) to describe salient characteristics of stroke patients with PCA infarction, b) to compare data of these patients with those with ischaemic stroke due to middle cerebral artery (MCA) and anterior cerebral artery (ACA) infarctions, and c) to identify predictors of PCA stroke.

## Methods

The database of the "Sagrat Cor Hospital of Barcelona Stroke Registry" with data of 3808 acute stroke patients was searched for those with a diagnosis of ischaemic stroke caused by occlusion in the territory of the PCA who were admitted consecutively to the Department of Neurology of the Sagrat Cor Hospital (an acute-care 350-bed teaching hospital in the city of Barcelona) between January 1986 and December 2004. Details of this on-going hospital-based stroke registry have been previously reported [4]. Classification of subtypes of stroke and definitions of vascular risk factors were those recommended by the Cerebrovascular Study Group of the Spanish Society of Neurology [5] and have been used in previous studies [4,6,7].

The objective of this clinical study was to assess differential features in aetiology, risk factors, clinical findings and early outcome between patients with PCA stroke and those with MCA and PCA infarctions. To this purpose, All patients with ischaemic cerebral infarction (n = 2704) included in the database were selected. The region of the infarction was identified on computerized tomographic (CT) scans and/or magnetic resonance imaging (MRI) studies and then PCA (*n *= 232), ACA (*n *= 51) and MCA (*n *= 1355) topographies were chosen. These three vascular territories were defined according to previously published and validated maps of cerebral vascular territories [8] and used in other studies [4,6,7,9]. Cortical, deep (involving thalamus and/or midbrain), or cortical and deep distribution in PCA topography were recorded. PCA infarcts with coincident infarcts outside the PCA territory were excluded (PCA plus infarctions) were excluded. Therefore, the study population included only patients with isolated PCA infarctions.

All patients were admitted to the hospital within 48 hours of onset of symptoms. On admission, demographic characteristics; salient features of clinical and neurological examination and results of laboratory tests (blood cell count, biochemical profile, serum electrolytes, urinalysis); chest radiography; twelve-lead electrocardiography. In all patients, brain CT scans were performed within the first week of hospital admission. Patients with negative results had a second CT during their stay in hospital or were studied by MRI (45.7%). Angio-MRI was obtained during hospitalisation in 33.3% of patients, echo-Doppler of the supra-aortic trunks in 60%, and arterial digital subtraction angiography in 9.8%. Other investigations included echocardiography in 56% of patients and lumbar puncture in 2%. The degree of clinical disability at discharge from the hospital was evaluated according to the Rankin scale until 1990 and with the modified Rankin scale (mRS) [10] from 1990 onwards. Causes of death were assesses according to the criteria of Silver et al. [11].

Prior to conducting the study, approval was obtained from the Ethical Committee of Clinical Research of Hospital Universitari Sagrat Cor. Consent was also obtained for the use of hospital data included in the Sagrat Cor Hospital of Barcelona Stroke Registry.

### Statistical analysis

Demographic characteristics, risk factors, clinical events and outcome of PCA stroke patients and those with infarctions of the MCA and ACA territories were compared using the Student's *t*-test or the Mann-Whitney *U *test for continuous variables and the chi-square (χ^2^) test (with Yate's correction when necessary) for categorical variables. Statistical significance was set at *P *< 0.05. Variables were subjected to multivariate analysis with a logistic regression procedure and forward stepwise selection if *P *< 0.10 after univariate testing. The effect of variables on the presence of infarction caused by occlusion of the PCA versus infarcts of the MCA and the ACA was studied in two multiple regression models based on demographic, vascular risk factors, and clinical and neuroimaging variables, in which the absence (codified as 0) or presence (codified as 1) of PCA infarction was the dependent variable. The level of significance was set as 0.15, and the tolerance level at 0.0001. The maximum likelihood approach was used to estimate weights of the logistic parameters. Odds ratio and 95% confidence intervals (CI) were calculated from the beta coefficients and standard errors. The hypothesis that the logistic model adequately fitted the data was tested by means of the goodness of fit χ^2 ^test. The SPSS-PC+ and the BMDP computer programmes were used for statistical analyses.

## Findings

The 232 patients PCA territory infarcts accounted for 6.1% of all cases of stroke (*n *= 3808) and 8.6% of cerebral infarction (*n *= 2704) included in the stroke registry. There were 128 men and 104 women with a mean (SD) age of 73.9 (11.9) years. Thirty-seven patients were 85 years or age or older. Vascular risk factors in a decreasing order of frequency included hypertension (58.6%), diabetes mellitus (30.2%), atrial fibrillation (26.7%) and dyslipidemia (19.4%). History of previous TIA was present in 12.9% of patients. Sudden onset of neurological deficit was recorded in 46.6% of cases. Sensory symptoms occurred in 51.3% of cases, hemianopia in 41.4%, limb weakness in 38.8 and speech disturbances (dysarthria, aphasia) in 30.2%. Memory disorders were recorded in 25%. Cortical territory involvement accounted in 51%, deep involvement in 35% and involvement of both cortical and deep in 14%. Stroke subtypes included lacunar infarct in 34.5%, atherothrombotic infarction in 29.3%, cardioembolic infarction in 21.6% of patients, infarct of unknown cause in 8.6% and infarction of unusual aetiology in 6%.

Nine patients died during the hospital stay, with an in-hospital mortality rate of 3.9%. Causes of death included herniation of the brain in 3, sudden death in 3, pneumonia in 2 and septicaemia in 1. The median length of hospitalization was 11 days (25th-75th percentile, 8-17 days). Forty-five patients (19.4%) were symptom-free at the time of hospital discharge (mRS grade 0-1). Of the remaining 187 patients, 58.6% had moderate disability (mRS grade 2-3), 17% moderately severe disability (mRS grade 4) and 5% severe disability (mRS grade 5).

As shown in Table 1, sex male, diabetes mellitus, headache at the time of stroke onset, dizziness, sensory symptoms, hemianopia, ataxia, symptom free at discharge and lacunar etiology were more frequent in stroke patients with PCA infarction than in those with MCA infarction and mean age, age > 85 years, atrial fibrillation, alcohol abuse, early seizures, speech disturbances, altered consciousness, limb weakness, and cardioembolic etiology were less frequent. On the other hand, headache at the time of stroke onset and sensory deficit were more frequent, and motor deficit and cardioembolism as the aetiology of stroke less frequent in patients with PCA infarction than in those with ACA infarction (Table 1).

**Table 1:** Results of univariate analysis

Variable	PCA	MCA	PCA *vs*. MCA *P *value	ACA	PCA *vs*. ACA *P *value
Total patients	232	1355		51	

Sex, male	128 (55.2)	635 (46.9)	0.019	27 (52.9)	0.445

Age, years, mean (SD)	73.9 (11.9)	76 (11.4)	0.011	74.4 (14.7)	0.801

Age ≥ 85 years	37 (15.9)	302 (22.3)	0.029	11 (21.6)	0.219

Risk factors					

Hypertension	136 (58.6)	769 (56.8)	0.595	28 (54.9)	0.369

Diabetes	70 (30.2)	301 (22.2)	0.008	15 (29.4)	0.530

Valvular Heart Disease	16 (6.9)	95 (7)	0.950	1 (2)	0.153

Coronary heart disease	36 (15.5)	223 (16.5)	0.720	4 (7.8)	0.111

Atrial fibrillation	62 (26.7)	461 (34)	0.029	12 (23.5)	0.391

Cardiac heart failure	9 (3.9)	88 (6.5)	0.124	4 (7.8)	0.190

Transient ischemic attack	30 (12.9)	152 (11.2)	0.449	4 (7.8)	0.225

Previous cerebral infarct	41 (17.7)	225 (16.6)	0.688	10 (19.6)	0.440

Previous hemorrhagic stroke	4 (1.7)	10 (0.7)	0.138	1 (2)	0.633

Peripheral vascular disease	13 (5.6)	100 (7.4)	0.331	2 (3.9)	0.472

Obesity	11 (4.7)	51 (3.8)	0.478	4 (7.8)	0.276

Alcohol abuse	1 (0.4)	39 (2.9)	0.028	0	0.820

Smoking (> 20 cigarettes/day)	20 (8.6)	132 (9.7)	0.592	3 (5.9)	0.376

Hyperlipidemia	45 (19.4)	226 (16.7)	0.309	9 (17.6)	0.474

Clinical findings					

Sudden onset	108 (46.6)	722 (53.3)	0.058	27 (52.9)	0.251

Headache	53 (22.8)	120 (8.9)	0.000	0	0.000

Dizzines	7 (3)	14 (1)	0.015	2 (3.9)	0.504

Early seazures	0	24 (1.8)	0.041	1 (2)	0.180

Nausea, vomiting	17 (7.3)	62 (4.6)	0.075	2 (3.9)	0.299

Aphasia and dysarthria	70 (30.2)	816 (60.2)	0.000	22 (43.1)	0.054

Altered consciousness	28 (12.1)	278 (20.5)	0.003	4 (7.8)	0.277

Limb weakness	90 (38.8)	1145 (84.5)	0.000	44 (86.3)	0.000

Sensory symptoms	119 (51.3)	554 (40.9)	0.003	15 (29.4)	0.003

Hemianopsia	96 (41.4)	264 (19.5)	0.000		

Ataxia	12 (5.2)	33 (2.4)	0.020		

Stroke Subtypes					0.001

Atherothrombotic	68 (29.3)	395 (29.2)		15 (29.4)	

Lacunar	80 (34.5)	301 (22.2)		6 (11.8)	

Cardioembolic	50 (21.6)	482 (35.6)		23 (45.1)	

Essential	20 (8.6)	124 (9.2)		6 (11.8)	

Unusual etiology	14 (6)	52 (3.9)		1 (2)	

Symptom-free at discharge	45 (19.4)	149 (11)	0.000	5 (9.8)	0.072

Cardiac complications	6 (2.6)	78 (5.8)	0.046	2 (3.9)	0.439

Infectious complications	15 (6.5)	237 (17.5)	0.000	6 (11.8)	0.155

Respiratory complications	12 (5.2)	176 (13)	0.001	4 (7.8)	0.321

Length of hospital stay, median (25th-75th percentile)	11 (8-17)	13 (9-23)	0.003	12 (10-24)	0.345

In-hospital mortality	9 (3.9)	235 (17.3)	0.000	4 (7.8)	0.190

Data are *n *(%) unless otherwise stated.

Comparison of patients with posterior cerebral artery infarction (PCA) with patients with middle cerebral artery infarction (MCA) and patients with anterior cerebral artery infarction (ACA)

In the multivariate analysis (Table 2), hemianopia (odds ratio [OR] = 6.43, 95% CI 4.25-9.74), lacunar as stroke mechanism (OR = 2.18, 95% CI 1.42-3.35), symptom-free at discharge (OR = 1.92, 95% CI 1.22-3.02), limb weakness (OR = 0.10, 95% CI 0.07-0.14), speech disorders (OR = 0.33, 95% CI 0.24-0.47) and cardioembolism as stroke mechanism (OR = 0.65, 95% CI 0.43-0.98) were independent variables of PCA stroke in comparison with MCA infarction, whereas sensory symptoms (OR = 2.36, 95% CI 1.16-4.82), limb weakness (OR = 0.11, 95% CI 0.04-0.25) and cardioembolism as stroke mechanism (OR = 0.43, 95% CI 0.21-0.85) were independent variables associated with PCA stroke as compared with ACA infarction.

**Table 2:** Results of multivariate analysis

Variable	β	SE (β)	Odds ratio (95% CI)	*P* value
PCA *versus*. MCA*				

Model based on demographic, vascular risk, clinical, and topographic data*				

Hemianopia	1.861	0.212	6.43 (4.25-9.74)	0.000

Lacunar infarction	0.780	0.219	2.18 (1.42-3.35)	0.000

Symptom-free at discharge	0.653	0.230	1.92 (1.22-3.02)	0.005

Limb weakness	-2.322	0.176	0.10 (0.07-0.14)	0.000

Speech disorders (dysarthria)	-1.102	0.175	0.33 (0.24-0.47)	0.000

Cardioembolic infarction	-0.436	0.214	0.65 (0.43-0.98)	0.042

PCA *versus *ACA				

Model based on demographic, vascular risk, clinical, and topographic data^†^				

Sensory symptoms	0.8610	0.363	2.36 (1.16-4.82)	0.018

Limb weakness	-2.258	0.437	0.11 (0.04-0.25)	0.000

Cardioembolic stroke	-0.856	0.352	0.43 (0.21-0.85)	0.015

*β = -0.526; SE (β) = 0.203; goodness-of-fit χ2 = 0.016; *df *= 8; *P *= 0.016. Area under the ROC curve = 0.828; sensitivity 71.6%; specificity 80.4%; VPP 38.5%, VPN 94.3%, correct classification 79.1%.

†β = 2,937; SE (β) = 0.429; goodness-of-fit χ2 = 9.751; *df *= 6; *P *= 0.136. Area under the ROC curve = 0.803; sensitivity 73.3%; specificity 64.7%; VPP 90.4%, VPN 34.7%, correct classification 71.7%.

## Discussion

According to data of our hospital-based stroke registry, cerebral infarctions in the PCA territory is a subgroup of unusual cerebral infarcts, accounting for 6.1% of all cases of stroke and 8.6% of all cases of cerebral infarctions. There is limited data on the frequency of cerebral infarctions in the territory of the PCA in the different clinical studies are scarce. Previous series of PCA infarcts varied between 34 patients in the study of Kinkel et al. [12) and 205 patients in the study of Lee et al. [13]. As shown in Table the present series of 232 patients is the largest group so far reported in the literature (Table 3).

**Table 3:** Main series of cerebral infarcts in the territory of the posterior cerebral artery (PCA) reported in the literature

First author, year [reference]	Patients	Most frequent clinical findings	Aetiology	Frequency total infarcts	Frequency total stroke	In-hospital mortality
Kinkel, 1984 [12]	34	Homonymous hemianopsia 76%, limb weakness 47%	NA	NA	NA	NA

Pessin, 1987 [19]	41*	Homonymous hemianopsia 61%, sensory deficits 20%, limb weakness 15%	Unknown 27%, cardioembolic 24%	NA	NA	5%

Servan, 1992 [23]	76	Homonymous hemianopsia 84%, sensory deficits 32%	Unknown 27%, cardioembolic 35%	NA	NA	NA

Bogousslavsky, 1993 [24]	70	NA	Large artery atherosclerosis 66%, cardio- embolism 21%, small-artery disease 16%	NA	NA	NA

Milandre, 1994 [21]	82	Homonymous hemianopsia 43%, sensory deficits 46%, limb weakness 34%	Unknown 11%, cardioembolic 18%	NA	4.2%	7%

Brandt, 1995 [26]	127	Homonymous hemianopsia 74%, sensory deficits 29%, limb weakness 28%	Unknown 20%, cardioembolic 28%	NA	NA	0%

Steinke, 1997 [22]	74*	Homonymous hemianopsia 62%, sensory deficits 15%, limb weakness 21%	Unknown 24%, intrinsic PCA disease 30% cardioembolic 31%	1.3%	1%	NA

Yamamoto, 1999 [18]	79	Homonymous hemianopsia 84%, sensory deficits 15%, limb weakness 29%	Proximal arterial disease 32% cardioembolism 41%	NA	NA	3%

Cals, 2002 [27]	117*	Hemianopsia 78%, limb weakness 12%, sensory deficits 12%	Unknown 32%, cardioembolism 43.5%	NA	3.5%	0%

Kumral, 2004 [20]	137		Intrinsic PCA disease 26%, proximal large artery disease 24%, cardioembolism 17%	NA	NA	7%

Lee, 2009 [13]	205		Atherosclerosis 42%, cardioembolic 20%	NA	NA	NA

Ntaios, 2011 [31]	185		Pure PCA^†^: cardioembolism 39%, unknown 22% PCA-plus^‡^: cardioembolism 63.4%	8.1% NA	8.2% 25.4%	NA

Present series, 2011	232	Homonymous hemianopsia 41%, sensory deficits 51%, limb weakness 39%	Lacunar 34.5%, atherosclerosis 29.3% cardioembolism 21.6%	8.6%	6.1%	3.9%

*Pure superficial PCA territory infarction.

^† ^Pure PCA: cortical or cortical and deep PCA infarcts.

^‡ ^PCA-plus: cortical or cortical and deep infarcts coincident with infarct(s) outside the PCA territory -posterior or anterior circulation.

NA: not applicable.

It should be noted that 15.9% of our patients were older than 85 years of age. This finding is consisted with results of other studies [14,15] and highlights the increasing prevalence and clinical relevance of first-ever stroke in the oldest old population segment of developed countries.

The aetiology of PCA infarctions is poorly defined in the literature, although in our study, the most frequent aetiopathogenic mechanism was lacunar in 34.5% of cases, followed by atherothrombosis in 29.3%, and cardioembolism in 21.6%. In 8.6% of the cases, the cause was unknown. The following criteria [5,16] were used to establish the lacunar origin of the infarction: (a) sudden or gradual onset of a focal neurological deficit lasting > 24 h of the type described in the common lacunar syndromes (pure motor hemiparesis, pure sensory stroke, sensorimotor stroke ataxia hemiparesis, dysarthria-clumsy hand, and atypical lacunar syndromes), (b) CT scans or brain MRI either normal or demonstrated only small, localized thalamic lesions with diameter smaller than 20 mm that seemed appropriate for the neurological deficits, and (c) absence of cortical ischaemia, cervical carotid stenosis (> 50% diameter) or major source for cardioembolic stroke. Cardioembolic stroke was defined [5,17] as a medium-to-large size cerebral infarction, usually of cortical topography in which in the absence of other aetiologies, some of the following emboligenous cardiac disorders are documented: atrial flutter or atrial fibrillation, intracardiac thrombus or tumour, rheumatic valve disease, mitral or aortic valve prosthesis, endocarditis, sinus node disease, left ventricular aneurysm after acute myocardial infarction, acute myocardial infarction in the acute phase (less than 3 months) or global cardiac hypokinesia.

This higher frequency of lacunar aetiology in PCA infarcts as compared with infarcts in the territory of the ACM (OR = 2.18) has not been previously documented, probably because not only the superficial PCA territory was considered in the analysis, but also the deep territory of thalamoperforating or thalamogeniculated penetrating arterioles, the occlusion of which causes subcortical lacunar infarcts of thalamic topography or mesencephalic involvement. These infarcts due to penetrating artery disease are usually included in the literature in the series of lacunar infarctions but are not specified in the series of infarctions of the PCA territory [18].

Cardioembolism as the aetiology of infarction was less frequent in PCA patients than in patients with ACM infarctions (OR = 0.65). The identification of cardioembolism in 21.6% of cases is similar to percentages of 24% reported in the study of Pessin et al. [19] and 20% in the study of Lee et al. [13] but clearly higher than 17% found in the study of Kumral et al. [20] and 18% in the study of Milandre et al. [21], and lower than 41% found by Yamamoto et al. [18], 31% by Steinke et al. [22] and 35% by Servan et al. [23]. In this respect, this aetiological aspect is important because a diagnosis of cardioembolism has practical implications in the management of these patients. In the presence of a cardioembolic infarction in the PCA territory, early anticoagulation at therapeutic doses as a secondary prevention of cardioembolic stroke should be indicated [24,25].

Patients with infarcts in the PCA territory have a relatively favourable short-term prognosis as shown by a higher frequency of symptom free at discharge (OR = 1.92) an in-hospital mortality rate of 3.9%, clearly lower that 17.3% of MCA infarctions. In the series of Brandt et al. [26] and Cals et al. [27], none of the patients with acute PCA territory infarcts died. Other authors have reported similar mortality rates (3% in the study of Yamamoto et al. [18]) or higher in-hospital mortality, such as 7% in the study of Milandre et al. [21], and 5% in the study of Pessin et al. [19].

It should be noted that PCA infarcts present a clinical profile clearly different than the remaining cerebral hemispheric infarctions. Hemianopia (OR = 6.43) was independent variable associated with PCA infarctions as compared with MCA stroke. Initial neurological deficits are predominantly visual followed by sensory complaints [8]. The lower frequency of dysarthria or aphasia is explained because both Broca's motor speech area and sensory speech area of Wernicke are located in the vascular territory of the MCA [1,8]. The lower frequency of limb weakness (OR = 0.10) is another clinical feature. Occurrence of motor deficits with PCA territory ischemia is considered unusual. Motor deficits was usually slight is due to oedema in the posterior limb of the internal capsule adjacent to the thalamus infarct with reversible impairment of pyramidal tracts. Hemiplegia in patients with PCA territory infarcts is usually caused by involvement of the cerebral peduncle [8].

Variables associated with PCA stroke *vs*. infarctions in the vascular territory of the ACA were sensory deficit, motor deficit, and cardioembolism. The higher frequency of sensory deficit in PCA stroke than in ACA infarction is explained because the deep vascular territory of the PCA includes the ventroposterolateral thalamic nucleus [28]. The frequency of motor deficit with a characteristic crural distribution is the most common neurological sign of ACA stroke and was present in 86.3% of our patients as compared with a prevalence of 93.3% in the series of Kumral et al. [29] and 96% in the experience of the Lausanne Stroke Registry [30]. Infarctions in the ACA territory usually involve the paracentral component of the frontal lobe affecting motor neurons with a somotatotopic distribution mostly related to the lower extremities [1].

There are no data on long-term outcome of PCA stroke. The recent study of Ntaios et al. [31] provides for the first time long-term follow-up data on prognosis in a large series of consecutive patients with infarctions in the PCA territory, showing that long-term mortality was associated with initial neurological severity (NIHSS score). This coincides with observations in the remaining ischaemic stroke subtypes in which motor weakness is an important clinical feature that causes greater focal neurological symptoms and is significantly associated with early death [10].

A recent study of intravenous thrombolysis in PCA infarcts has shown no substantial differences regarding baseline stroke severity, outcome, safety and clinical findings between supratentorial PCA infarct patients and all patients with acute ischaemic stroke which would implicate a change in the existing thrombolysis practice in patients with PCA stroke [32].

Finally, a limitation of the study is related to the fact that not all patients underwent angio MR or CT angiogram to exclude the possibility of PCA stenosis, so that some of the patients with lacunar infarctions may in fact have PCA stenosis, and in these circumstances in the narrow sense, may be considered as atherotrhrombotic infarcts or infarcts of unknown cause by the presence of a double etiological mechanism: lacunar and atherotrhrombotic.

## Conclusions

Cerebral infarcts in the PCA territory are infrequent and account for only 6.1% of all cases of cerebral infarction and 8.6% of all cases of stroke. Lacunar infarction is the main aetiological ischaemic subtype of PCA stroke. Patients with PCA infraction have a favourable short-term prognosis. In-hospital mortality in patients with PCA territory is low and shows a clinical profile different than the remaining cerebral hemispheric infarcts.

## Abbreviations

ACA: anterior cerebral artery; CI: Confidence interval; CT: Computed tomography; MCA: middle cerebral artery; MRI: Magnetic resonance imaging; OR: Odds ratio; PCA: Posterior cerebral artery; ROC: Receiver operating characteristics; SD: standard deviation; TIA: Transient ischaemic attack.

## Competing interests

The authors declare that they have no competing interests.

## Authors' contributions

AA was the principal investigator, designed the study, diagnosed and took care of the patients, contributed to analyze the data, interpreted the results, wrote the paper, and prepared the final draft. He was also responsible for editorial decisions including the selection of the journal GA, MO, OP and JM participated in the collection of data medical care of the patients, analysis of results, and review of the manuscript for intellectual content. LGE was the statistician, participated in the study design, analysis and interpretation of data, and wrote the part of the paper related to the statistical analysis. All authors read and approved the final manuscript.
